# On the concept of the normative in the assessment of mental disorder

**DOI:** 10.3389/fpsyg.2014.00129

**Published:** 2014-02-17

**Authors:** Sebastian Muders

**Affiliations:** Department of Philosophy, Center for Ethics, University of ZurichZurich, Switzerland

**Keywords:** concept of mental disorder, non-natural, objectivity, relativity, normativity

In Marco Stier's article “Normative preconditions for the assessment of mental disorder,” the concept of the normative occupies a central role (Stier, 2013). Stier states that mental disorders have an irreducible normative element built in, expressible through various “normative frames of reference” they are tied to. Following his two main theses, he thinks that these frameworks shape what counts as deviant as well as non-deviant behavior. He takes this as evidence that we have to specify mental disorders at the mental level, and thus will never be able to give a purely physical account of them.

Unfortunately, he nowhere makes clear what he takes to be the content of the concept of the normative, although he gives some hints about his understanding at various passages. In what follows, I will explore three of his implicit suggestions on the essential linkages his concept of the normative bears to other concepts: the non-natural, the non-objective, and the relative. I shall argue that it is questionable that this understanding leads to the conclusion Stier aims at—that the specification of mental disorders cannot be succeed on the physical but only the mental level due to the impact of normative considerations in this enterprise.

## The normative and the non-natural

Regarding the relationship between the normative and the non-natural, Stier argues that the normative cannot be grasped in naturalistic terms. Integrating this alleged fact into his ontological dichotomy between the mental and the natural, it follows for him that the normative must belong to the realm of the mental, for “[t]here seems to be something peculiar about behavior that is beyond purely physical explanation because the difference between, say, acting kindly and unkindly can hardly be grasped in physical, non-normative terms” (p. 1).

Both thoughts appear to be problematic. With respect to the first, there is a whole bunch of philosophers out there that intend to explicate all kinds of normative facts related to human behavior in naturalistic terms, therefore reducing the normative to the natural. What Derek Parfit calls “Analytical Naturalism” (cf. Parfit, 2011, p. 295) precisely aims at re-defining normative notions in terms of natural notions. Parfit mentions Nicholas Sturgeon and Frank Jackson as prominent proponents of this type of naturalism (Parfit, 2011, p. 365).

Whether these people are right or wrong is certainly subject to discussion, but their efforts at least suggest that taking phenomena at face value and not even mentioning competing accounts can hardly be the adequate strategy. Indeed, Stier himself gives a prominent example of a *prima facie* irreducible ontological domain besides the physical: The mental itself is often seen as an important challenge for the hard-boiled naturalist. Nevertheless, Stier grants (at least for the purpose of his paper) that “every single aspect of our mental and behavioral life could be explained in purely physical terms” (p. 2)—from which he of course explicitly excludes mental disorders. But if the vast majority of mental phenomena could be explained in natural terms—why should not the same be possible for normative ones?

Secondly, there seems to be no intuitive way for marrying the fact that something is non-natural with the fact that it is mental. A great many deal of things that may serve as paradigmatic examples for non-natural entities are not perceived as necessarily being mental in the way that is of interest here: God, the number seven, or human dignity—the latter being clearly a *normative* idea. God, for instance, is almost by definition non-natural, but his concept surely does not demand from us that he has to be imagined as something mental.

Indeed, that a certain normative entity cannot be grasped in natural terms does not mean that it has to be reducible to any other domain at all. Alternatively, the normative aspects of mental disorders that prove not to be analyzable in natural terms can be just that—irreducible normative aspects. For large parts of the normative domain, this is a common option. Russ Shafer-Landau, for example, argues that we should “introduce into our ontology a *sui generis* category of values” that can explain the normativity found in morality (cf. Shafer-Landau, 2003: 55). If one succeeds in providing a similar account for the normative aspects of mental disorders, there is no need to suppose that mental disorders have to specified at the mental level.

## The normative and the non-objective

Let us consider next the non-objective. When analyzing the various sorts of normative frames of reference, Stier frequently uses his assumption of a certain fact's being normative as a reason that is must be non-objective.

I will confine myself to two examples. When investigating evaluations of rationality and their bearing on the attribution of mental disorders, he states that “[i]t is [&] a matter of normative choice and not one of objective judgment whether rationality is regarded as a component of mental health or not” (p. 4). Since we often use irrational behavior as an indicator for the presence of a mental disorder, the attribution of the latter, if based on the former, also becomes a non-objective judgment.

One of Stier's arguments for this verdict is based on the general observation that one and the same person can be subject to a different treatment by others and the authorities, depending on whether a certain irrational behavior by her is regarded as due to a mental disorder or not. Thus, according to Stier's “Switching-Standard-Thesis,” judgments of rationality are not an “objective” standard for measuring one's mental health.

But this argument fails, for all Stier's observation shows is that depending on the supposed source of the irrational behavior (which might be subject to an argument from the best explanation), our reaction varies: If we have reasons to think that the person's irrational behavior is due to a psychological urge she does not recognize as part of her own personality, we are rightly more eager to intervene. Therefore, it is not as if the normative judgment about the person's rationality does change its validity from “true” to “false,” or as if the fact that she behaved irrational is somehow ontologically dependent on subjective elements; the change is only in the practical reasons both provide us with. And we have heard no argument that these reasons are not objective, only that their content depends on further considerations whose status is yet unclear.

Stier discovers an analogous fault when moral considerations are used for the assessing of mental health: “I do claim [&] that many conditions [of psychiatric disorders]—or conditions in many circumstances—at least involve (morally) normative elements and thus cannot be purely value free, non-normative (objective) medical kinds” (p. 5).

Stier's assumption that many kinds of mental disorders have a moral evaluation built in and are thus not “objective” in the sense of “out there independently of our subjective evaluation” seems to confuse people's opinions about the right morality with morality itself. He writes, for instance, that “[i]n a strictly religious society being an atheist may be seen as a dysfunction of personhood” (p. 5). True – but unless one subscribes to a flat subjectivism of the form “thinking that something is right makes it right,” *opinions* about morality are not necessarily true. Consequently, a diagnosed mental disorder partly based on observed moral misbehavior might be just false instead of arbitrarily (but nevertheless correctly) attributable.

## The normative and the relative

Finally, I want to take a look at Stier's assumed relationship between the normative and the relative. He uses the concept of the relative at various places and cites it as evidence that the value laden nature of mental disorder cannot be regarded as open to a purely natural analysis of the former. In sum, he states that “[p]sychiatry is guided by social, moral, cultural and other norms. If this is true, and if it is also true that these kinds of norms are relative to time and place, then psychiatry cannot claim to know what a mental disorder is “in itself,” where normality ends and mental disorder begins” (p. 3).

One passage where he substantiates this last quotation is his explication of the influence of *cultural* relativity for what counts as mental disorder. He distinguishes a direct from an indirect form of this influence. The former refers to the “cultural setup” of a society that creates norms its individuals have to follow. Depending on their personality, individuals might find it very difficult to cope with the expectations expressed in these norms and thus receive higher scores on certain criteria for specific psychological disorders.

That the frequency of the occurrence of psychological disorders correlates with cultural character and personality is hardly surprising and “relative” only in the most uninteresting sense of the term. It is a well-known fact that water's boiling point is dependent on a number of factors, including air pressure. This does not prevent it from being a perfect natural as well as objective fact so far. And neither cultural character nor personality is something that can be influenced at will by the individual to any degree relevant for most psychological disorders. Following this reasoning, Stier's demand that psychological problems have to be “all the same around the world” to receive a scientific explanation (cf. p. 7) is far too exaggerated.

With “direct influence,” Stier points to the thesis that culture characteristics may not only trigger the development of mental disorders in people with certain personalities; it also “tends to dictate the boundary between the normal and the deviant on the basis of the expected values and virtues of its members” (p. 6). Again, the example Stier uses makes only sense when assuming that there is no way to evaluate the criteria put forward for mental disorder in a given culture in terms of their appropriateness. That “[s]omebody who is “dynamic” in one cultural region may be regarded as offensive in another” (p. 7) just does not tell us whether she indeed *should be regarded* as offensive.

Stier thinks that there exists no “final answer” to this question, stating that different models of explanation in psychology all constitute “a basic explanatory *norm*” for determining what a mental disorder is. Thus, “there just is no higher level of objectivity from which we could assess the validity of one explanatory account or the other” (p. 7). To justify this bold thesis, he argues that even seemingly “hard” criteria such as the “effectiveness of an explanation and its respective therapies” cannot provide us with a solution, since which of the competing models of explanation “are the most effective ones is open to debate even today” (p. 8). However, the fact that a debate is still in an ongoing state as such is no legitimate criterion for thinking that it never can be closed via rational exchange, as proponents of the argument from relativity already admitted 30 years ago (Mackie, 1977, 36f.).

In sum, seeing mental disorders as having certain “normative aspects” does neither have to mean that they are only explicable at the level of the mental, nor that they cannot be stated objectively, nor that they exist or are recognizable only relative to cultural norms. The failure to provide a throughout “natural” characterization of them may be completely compatible with the view that they are, as Stier puts it, “out there” and have to be discovered rather than construed. And inasmuch not every normative aspect may be in need of being reducible to the natural, the mental, or anything else, nothing might be lost in translation.
